# Supplementary material for: B-Cell Epitope Mapping of TprC and TprD Variants of Treponema pallidum Subspecies Informs Vaccine Development for Human Treponematoses
Source: Front Immunol. 2022 Mar 29;13:862491. doi: 10.3389/fimmu.2022.862491 (PMC9001972; doi:10.3389/fimmu.2022.862491)
Supplement: Supplementary file 2 [file Table_2.docx]

| **Table S6.** Synthetic peptides used in this study. Amino acids encompassing ExLs (predicted by AlphaFold) are highlighted in red with yellow text. | | | | | | | | | | | | | | | | | | | |
| --- | --- | --- | --- | --- | --- | --- | --- | --- | --- | --- | --- | --- | --- | --- | --- | --- | --- | --- | --- |
|  |  | **Percentage Identity with Peptide by Strain and Allele*** | | | | | | | | | | | | | | | | | |
|  |  | **Nichols** | | **Chicago** | | **Bal73-1** | | **MexicoA** | | **Sea81-4** | | **Bal3** | | **UW249** | | **SamoaD** | | **IraqB** | |
| **Name** | **Peptide Sequence** | **Tpr**  **C** | **Tpr**  **D** | **Tpr**  **C** | **Tpr**  **D** | **Tpr**  **C** | **Tpr**  **D** | **Tpr**  **C** | **Tpr**  **D** | **Tpr**  **C** | **Tpr**  **D** | **Tpr**  **C** | **Tpr**  **D** | **Tpr**  **C** | **Tpr**  **D** | **Tpr**  **C** | **Tpr**  **D** | **Tpr**  **C** | **Tpr**  **D** |
| C1 | GVLTPQVSGTAQLQWGIAFQ | 100 | 100 | 100 | 100 | 100 | 100 | 100 | 100 | 100 | 100 | 100 | 100 | 100 | 100 | 100 | 100 | 100 | 100 |
| C2 | AQLQWGIAFQKNPRTGPGKH | 100 | 100 | 100 | 100 | 100 | 100 | 100 | 100 | 100 | 100 | 100 | 100 | 100 | 100 | 90 | 90 | 90 | 90 |
| C2 (SamoaD/IraqB) | AQLQWGIAFQKNPHTVPGKH | 90 | 90 | 90 | 90 | 90 | 90 | 90 | 90 | 90 | 90 | 90 | 90 | 90 | 90 | 100 | 100 | 100 | 100 |
| C3 | KNPRTGPGKHTHGFRTTNSL | 100 | 100 | 100 | 100 | 100 | 100 | 100 | 100 | 100 | 100 | 100 | 100 | 100 | 100 | 90 | 90 | 90 | 90 |
| C3 (SamoaD/IraqB) | KNPHTVPGKHTHGFRTTNSL | 90 | 90 | 90 | 90 | 90 | 90 | 90 | 90 | 90 | 90 | 90 | 90 | 90 | 90 | 100 | 100 | 100 | 100 |
| C4 | THGFRTTNSLTISLPLVSKH | 100 | 100 | 100 | 100 | 100 | 100 | 100 | 100 | 100 | 100 | 100 | 100 | 100 | 100 | 100 | 100 | 100 | 100 |
| C5 | TISLPLVSKHTHTRRGEARS | 100 | 100 | 100 | 100 | 100 | 100 | 100 | 100 | 100 | 100 | 100 | 100 | 100 | 100 | 100 | 100 | 100 | 100 |
| C6 | THTRRGEARSGVWAQLQLKD | 100 | 100 | 100 | 100 | 100 | 100 | 100 | 100 | 100 | 100 | 100 | 100 | 100 | 100 | 100 | 100 | 100 | 100 |
| C7 | GVWAQLQLKDLAVELASSKS | 100 | 100 | 100 | 100 | 100 | 100 | 100 | 100 | 100 | 100 | 100 | 100 | 100 | 100 | 100 | 100 | 100 | 100 |
| C8 | LAVELASSKSSTALSFTKPT | 100 | 100 | 100 | 100 | 100 | 100 | 100 | 100 | 100 | 100 | 100 | 100 | 100 | 100 | 100 | 100 | 100 | 100 |
| C9 | STALSFTKPTASFQATLHCY | 100 | 100 | 100 | 100 | 100 | 100 | 100 | 100 | 100 | 100 | 100 | 100 | 100 | 100 | 100 | 100 | 100 | 100 |
| C10 | ASFQATLHCYGAYLTVGTSP | 100 | 100 | 100 | 100 | 100 | 100 | 100 | 100 | 100 | 100 | 100 | 100 | 100 | 100 | 100 | 100 | 100 | 100 |
| C11 | GAYLTVGTSPSCVVNFAQLW | 100 | 100 | 100 | 100 | 100 | 100 | 100 | 100 | 100 | 100 | 100 | 100 | 100 | 100 | 100 | 100 | 100 | 100 |
| C12 | SCVVNFAQLWKPFVTRAYSE | 100 | 100 | 100 | 100 | 100 | 100 | 100 | 100 | 100 | 100 | 100 | 100 | 100 | 100 | 100 | 100 | 100 | 100 |
| C13 | KPFVTRAYSEKDTRYAPGFS | 100 | 100 | 100 | 100 | 100 | 100 | 100 | 100 | 100 | 100 | 100 | 100 | 100 | 100 | 100 | 100 | 100 | 100 |
| C14 | KDTRYAPGFSGSGAKLGYQA | 100 | 100 | 100 | 100 | 100 | 100 | 100 | 100 | 100 | 100 | 100 | 100 | 100 | 100 | 100 | 100 | 100 | 100 |
| C15 | GSGAKLGYQAHNVGNSGVDV | 100 | 100 | 100 | 100 | 100 | 100 | 100 | 100 | 100 | 100 | 100 | 100 | 100 | 100 | 100 | 100 | 100 | 100 |
| C16 | HNVGNSGVDVDIGFLSFLSN | 100 | 100 | 100 | 100 | 100 | 100 | 100 | 100 | 100 | 100 | 100 | 100 | 100 | 100 | 100 | 100 | 100 | 100 |
| C17 | DIGFLSFLSNGAWDSTDTTH | 100 | 100 | 100 | 100 | 100 | 100 | 100 | 100 | 100 | 100 | 100 | 100 | 100 | 100 | 100 | 100 | 100 | 100 |
| C18 | GAWDSTDTTHSKYGFGADAT | 100 | 100 | 100 | 100 | 100 | 100 | 100 | 100 | 100 | 100 | 100 | 100 | 100 | 100 | 100 | 100 | 100 | 100 |
| C19 | SKYGFGADATLSYGVDRQRL | 100 | 100 | 100 | 100 | 100 | 100 | 100 | 100 | 100 | 100 | 100 | 100 | 100 | 100 | 100 | 100 | 100 | 100 |
| C20 | LSYGVDRQRLLTLELAGNAT | 100 | 100 | 100 | 100 | 100 | 100 | 100 | 100 | 100 | 100 | 100 | 100 | 100 | 100 | 100 | 100 | 100 | 100 |
| C21 | LTLELAGNATLDQNYVKGTE | 100 | 100 | 100 | 100 | 100 | 100 | 85 | 100 | 100 | 100 | 100 | 100 | 100 | 100 | 85 | 85 | 90 | 90 |
| C21 (MexicoA/SamoaD) | LTLELAGNATLEQHYRKGTE | 85 | 85 | 85 | 85 | 85 | 85 | 100 | 85 | 85 | 85 | 85 | 85 | 85 | 85 | 100 | 100 | 90 | 90 |
| C21 (IraqB) | LTLELAGNATLEQNYLKGTE | 90 | 90 | 90 | 90 | 90 | 90 | 90 | 90 | 90 | 90 | 90 | 90 | 90 | 90 | 90 | 90 | 100 | 100 |
| C22 | LDQNYVKGTEDSKNENKTAL | 100 | 100 | 100 | 100 | 100 | 100 | 80 | 100 | 100 | 100 | 100 | 100 | 100 | 100 | 80 | 80 | 85 | 85 |
| C22 (MexicoA/SamoaD) | LEQHYRKGTEDSTNENKTAL | 80 | 80 | 80 | 80 | 80 | 80 | 100 | 80 | 80 | 80 | 80 | 80 | 80 | 80 | 100 | 100 | 80 | 80 |
| C22 (IraqB) | LEQNYLKGTEDPKNENKTAL | 85 | 85 | 85 | 85 | 85 | 85 | 80 | 85 | 85 | 85 | 85 | 85 | 85 | 85 | 80 | 80 | 100 | 100 |
| C23 | DSKNENKTALLWGVGGRLTL | 100 | 100 | 100 | 100 | 100 | 100 | 95 | 100 | 100 | 100 | 100 | 100 | 100 | 100 | 95 | 95 | 95 | 95 |
| C23 (MexicoA/SamoaD) | DSTNENKTALLWGVGGRLTL | 95 | 95 | 95 | 95 | 95 | 95 | 100 | 95 | 95 | 95 | 95 | 95 | 95 | 95 | 100 | 100 | 90 | 90 |
|  |  | **Percentage Identity with Peptide by Strain and Allele** | | | | | | | | | | | | | | | | | |
|  |  | **Nichols** | | **Chicago** | | **Bal73-1** | | **MexicoA** | | **Sea81-4** | | **Bal3** | | **UW249** | | **SamoaD** | | **IraqB** | |
| **Name** | **Peptide Sequence** | **Tpr**  **C** | **Tpr**  **D** | **Tpr**  **C** | **Tpr**  **D** | **Tpr**  **C** | **Tpr**  **D** | **Tpr**  **C** | **Tpr**  **D** | **Tpr**  **C** | **Tpr**  **D** | **Tpr**  **C** | **Tpr**  **D** | **Tpr**  **C** | **Tpr**  **D** | **Tpr**  **C** | **Tpr**  **D** | **Tpr**  **C** | **Tpr**  **D** |
| C23 (IraqB) | DPKNENKTALLWGVGGRLTL | 95 | 95 | 95 | 95 | 95 | 95 | 90 | 95 | 95 | 95 | 95 | 95 | 95 | 95 | 90 | 90 | 100 | 100 |
| C24 | LWGVGGRLTLEPGAGFRFSF | 100 | 100 | 100 | 100 | 100 | 100 | 100 | 100 | 100 | 100 | 100 | 100 | 100 | 100 | 100 | 100 | 100 | 100 |
| C25 | EPGAGFRFSFALDAGNQHQS | 100 | 100 | 100 | 100 | 100 | 100 | 100 | 100 | 100 | 100 | 100 | 100 | 100 | 100 | 100 | 100 | 100 | 100 |
| C26 | ALDAGNQHQSNAHAQTQERA | 100 | 100 | 100 | 100 | 100 | 100 | 100 | 60 | 100 | 60 | 100 | 60 | 100 | 60 | 85 | 50 | 85 | 50 |
| C26 (IraqB) | ALDAGNQHQSNADAQTQEER | 85 | 85 | 85 | 85 | 85 | 85 | 85 | 60 | 85 | 60 | 85 | 60 | 85 | 60 | 95 | 50 | 100 | 50 |
| C26 (SamoaD) | ALDAGNQHQSNAHAQTQKER | 80 | 80 | 80 | 80 | 80 | 80 | 80 | 60 | 80 | 60 | 80 | 60 | 80 | 60 | 100 | 50 | 95 | 50 |
| C27 | NAHAQTQERAILKAREVFRR | 100 | 100 | 100 | 100 | 100 | 100 | 100 | 20 | 100 | 20 | 100 | 20 | 100 | 10 | 40 | 20 | 55 | 20 |
| C27 (IraqB) | NADAQTQEERVSLAGEVFGQ | 55 | 55 | 55 | 55 | 55 | 55 | 55 | 20 | 55 | 20 | 55 | 20 | 55 | 20 | 90 | 10 | 100 | 10 |
| C27 (SamoaD) | NAHAQTQKERVSLAGEVFGQ | 65 | 50 | 65 | 65 | 50 | 65 | 65 | 10 | 65 | 10 | 65 | 10 | 65 | 10 | 100 | 10 | 90 | 10 |
| C28 | ILKAREVFRRVEGKLVQNLP | 100 | 100 | 100 | 100 | 100 | 100 | 100 | 15 | 100 | 15 | 100 | 15 | 100 | 15 | 65 | 15 | 65 | 15 |
| C28 (SamoaD/IraqB) | VSLAGEVFGQVVGKLVQNLP | 65 | 65 | 65 | 65 | 65 | 65 | 65 | 15 | 65 | 15 | 65 | 15 | 65 | 15 | 100 | 15 | 100 | 15 |
| C29 | VEGKLVQNLPNIMMPPGITE | 100 | 100 | 100 | 100 | 100 | 100 | 100 | 5 | 100 | 5 | 100 | 5 | 100 | 5 | 90 | 5 | 90 | 5 |
| C29 (SamoaD/IraqB) | VVGKLVQNLPNIMMPLGITE | 90 | 90 | 90 | 90 | 90 | 90 | 90 | 5 | 90 | 5 | 90 | 5 | 90 | 5 | 100 | 5 | 100 | 5 |
| C30 | NIMMPPGITEQTTLIEMVGL | 100 | 100 | 100 | 100 | 100 | 100 | 100 | 20 | 100 | 20 | 100 | 20 | 100 | 20 | 95 | 20 | 95 | 20 |
| C31 | QTTLIEMVGLAALIAEGTLG | 100 | 100 | 100 | 100 | 100 | 100 | 100 | 15 | 100 | 15 | 100 | 15 | 100 | 15 | 95 | 15 | 95 | 15 |
| C32 | AALIAEGTLGSAIQTVLAAG | 100 | 100 | 100 | 100 | 100 | 100 | 100 | 15 | 100 | 15 | 100 | 15 | 100 | 15 | 100 | 15 | 100 | 15 |
| C33 | SAIQTVLAAGALAALVSQLV | 100 | 100 | 100 | 100 | 100 | 100 | 100 | 10 | 100 | 10 | 100 | 10 | 100 | 10 | 100 | 10 | 100 | 10 |
| C34 | ALAALVSQLVPNIEQGVRDV | 100 | 100 | 100 | 100 | 100 | 100 | 100 | 5 | 100 | 5 | 100 | 5 | 100 | 5 | 95 | 5 | 95 | 5 |
| C34 (SamoaD/IraqB) | ALAALVSQLVPHIEQGVRDV | 95 | 95 | 95 | 95 | 95 | 95 | 95 | 5 | 95 | 5 | 95 | 5 | 95 | 5 | 100 | 5 | 100 | 5 |
| C35 | PNIEQGVRDVFRSSDPRVVT | 100 | 100 | 100 | 100 | 100 | 100 | 100 | 20 | 100 | 20 | 100 | 20 | 100 | 20 | 95 | 20 | 95 | 20 |
| C35 (SamoaD/IraqB) | PHIEQGVRDVFRSSDPRVVT | 95 | 95 | 95 | 95 | 95 | 95 | 95 | 20 | 95 | 20 | 95 | 20 | 95 | 20 | 100 | 20 | 100 | 20 |
| C36 | FRSSDPRVVTAKLLAFLERA | 100 | 100 | 100 | 100 | 100 | 100 | 100 | 25 | 100 | 25 | 100 | 25 | 100 | 25 | 100 | 25 | 100 | 25 |
| C37 | AKLLAFLERAPMNALNIDAL | 100 | 100 | 100 | 100 | 100 | 100 | 100 | 40 | 100 | 40 | 100 | 40 | 100 | 40 | 100 | 40 | 100 | 40 |
| C38 | PMNALNIDALLRMQWKWLSS | 100 | 100 | 100 | 100 | 100 | 100 | 100 | 75 | 100 | 75 | 100 | 75 | 100 | 75 | 100 | 70 | 100 | 80 |
| C39 | LRMQWKWLSSGIYFATAGTN | 100 | 100 | 100 | 100 | 100 | 100 | 100 | 95 | 100 | 95 | 100 | 95 | 100 | 95 | 100 | 90 | 100 | 100 |
| C40 | GIYFATAGTNIFGKRVFATT | 100 | 100 | 100 | 100 | 100 | 100 | 100 | 80 | 100 | 80 | 100 | 80 | 100 | 80 | 100 | 80 | 100 | 80 |
| C41 | IFGKRVFATTRAHYFDFAGF | 100 | 100 | 100 | 100 | 100 | 100 | 100 | 60 | 100 | 60 | 100 | 60 | 100 | 60 | 100 | 70 | 100 | 70 |
| C42 | RAHYFDFAGFLKLETKSGDP | 100 | 100 | 100 | 100 | 100 | 100 | 100 | 80 | 100 | 80 | 100 | 80 | 100 | 80 | 100 | 80 | 100 | 80 |
| C43 | LKLETKSGDPYTHLLTGLNA | 100 | 100 | 100 | 100 | 100 | 100 | 100 | 100 | 100 | 100 | 100 | 100 | 100 | 100 | 100 | 100 | 100 | 100 |
|  |  | **Percentage Identity with Peptide by Strain and Allele** | | | | | | | | | | | | | | | | | |
|  |  | **Nichols** | | **Chicago** | | **Bal73-1** | | **MexicoA** | | **Sea81-4** | | **Bal3** | | **UW249** | | **SamoaD** | | **IraqB** | |
| **Name** | **Peptide Sequence** | **Tpr**  **C** | **Tpr**  **D** | **Tpr**  **C** | **Tpr**  **D** | **Tpr**  **C** | **Tpr**  **D** | **Tpr**  **C** | **Tpr**  **D** | **Tpr**  **C** | **Tpr**  **D** | **Tpr**  **C** | **Tpr**  **D** | **Tpr**  **C** | **Tpr**  **D** | **Tpr**  **C** | **Tpr**  **D** | **Tpr**  **C** | **Tpr**  **D** |
| C44 | YTHLLTGLNAGVEARVYIPL | 100 | 100 | 100 | 100 | 100 | 100 | 100 | 100 | 100 | 100 | 100 | 100 | 100 | 100 | 100 | 100 | 100 | 100 |
| C45 (Nichols) | GVEARVYIPLTYIRYRNNGG | 100 | 100 | 100 | 100 | 100 | 100 | 90 | 95 | 90 | 95 | 90 | 95 | 90 | 90 | 90 | 95 | 90 | 95 |
| C45 (MexicoA/Sea814/  Bal3/UW249) | GVEARVYIPLTYVFYRNNGG | 90 | 90 | 90 | 90 | 90 | 90 | 100 | 90 | 100 | 90 | 100 | 90 | 100 | 90 | 100 | 90 | 100 | 90 |
| C45 (SamoaD/IraqB) | GVEARVYIPLTYVFYKNNGG | 90 | 90 | 90 | 90 | 90 | 90 | 100 | 95 | 100 | 95 | 95 | 95 | 100 | 95 | 100 | 95 | 100 | 95 |
| C46 (Nichols) | TYIRYRNNGGYELNGAVPPG | 100 | 100 | 100 | 100 | 100 | 100 | 75 | 50 | 75 | 50 | 75 | 50 | 75 | 50 | 70 | 45 | 80 | 45 |
| C46 (MexicoA/Sea814/  Bal3/UW249) | TYVFYRNNGGYELNRVVPSG | 75 | 75 | 75 | 75 | 75 | 75 | 100 | 45 | 100 | 45 | 100 | 45 | 100 | 45 | 85 | 45 | 85 | 45 |
| C46 (IraqB) | TYVFYKNNGGYELNGVVPPG | 80 | 80 | 80 | 80 | 80 | 80 | 100 | 45 | 100 | 45 | 95 | 45 | 100 | 45 | 90 | 50 | 100 | 50 |
| C46 (SamoaD) | TYVFYKNNGGYPLNGVVPSG | 70 | 70 | 70 | 70 | 70 | 70 | 80 | 50 | 80 | 50 | 80 | 50 | 85 | 50 | 100 | 50 | 90 | 50 |
| C47 (Nichols) | YELNGAVPPGTINMPILGKA | 100 | 100 | 100 | 100 | 100 | 100 | 80 | 45 | 80 | 45 | 80 | 45 | 80 | 45 | 85 | 45 | 90 | 45 |
| C47 (MexicoA/Sea814/  Bal3/UW249) | YELNRVVPSGIINMPILGKA | 80 | 80 | 80 | 80 | 80 | 80 | 100 | 40 | 100 | 40 | 100 | 40 | 100 | 40 | 85 | 45 | 90 | 45 |
| C47 (IraqB) | YELNGVVPPGIINMPILGKA | 75 | 75 | 75 | 75 | 75 | 75 | 85 | 50 | 85 | 50 | 85 | 50 | 85 | 50 | 85 | 50 | 100 | 50 |
| C47 (SamoaD) | YPLNGVVPSGTINMPILGKA | 85 | 85 | 85 | 85 | 85 | 85 | 85 | 50 | 85 | 50 | 85 | 50 | 85 | 50 | 100 | 50 | 85 | 50 |
| C48 (Nichols/SamoaD) | TINMPILGKAWCSYRIPLGS | 100 | 100 | 100 | 100 | 100 | 100 | 95 | 85 | 95 | 85 | 95 | 85 | 95 | 85 | 100 | 85 | 95 | 85 |
| C48 (MexicoA/Sea814/  Bal3/UW249/IraqB) | IINMPILGKAWCSYRIPLGS | 95 | 95 | 95 | 95 | 95 | 95 | 100 | 85 | 100 | 85 | 100 | 85 | 100 | 85 | 95 | 85 | 100 | 85 |
| C49 | WCSYRIPLGSHAWLAPHTSV | 100 | 100 | 100 | 100 | 100 | 100 | 100 | 100 | 100 | 100 | 100 | 100 | 100 | 100 | 100 | 100 | 100 | 100 |
| C50 | HAWLAPHTSVLGTTNRFNII | 100 | 100 | 100 | 100 | 100 | 100 | 100 | 100 | 100 | 100 | 100 | 100 | 100 | 100 | 100 | 100 | 100 | 100 |
| C51 (Nichols) | LGTTNRFNIINPAGNLLNER | 100 | 100 | 100 | 100 | 100 | 100 | 95 | 45 | 95 | 45 | 95 | 45 | 95 | 45 | 90 | 40 | 90 | 40 |
| C51 (MexicoA/ Sea81-4/Bal3/UW249) | LGTTNRFNIINAAGNLLNER | 95 | 95 | 95 | 95 | 95 | 95 | 100 | 45 | 100 | 45 | 100 | 45 | 100 | 45 | 95 | 45 | 95 | 45 |
| C51 (SamoaD/IraqB) | LGTTNRFNIINAAGNLVNER | 90 | 90 | 90 | 90 | 90 | 90 | 95 | 45 | 95 | 45 | 95 | 45 | 95 | 45 | 100 | 40 | 100 | 40 |
| C52 (Nichols) | NPAGNLLNERALQYQVGLTF | 100 | 100 | 100 | 100 | 100 | 100 | 95 | 65 | 95 | 65 | 95 | 65 | 95 | 65 | 90 | 60 | 90 | 60 |
| C52 (MexicoA/ Sea81-4/Bal3/UW249) | NAAGNLLNERALQYQVGLTF | 95 | 95 | 95 | 95 | 95 | 95 | 100 | 65 | 100 | 65 | 100 | 65 | 100 | 65 | 95 | 60 | 95 | 60 |
| C52 (SamoaD/IraqB) | NAAGNLVNERALQYQVGLTF | 90 | 90 | 90 | 90 | 90 | 90 | 95 | 60 | 95 | 60 | 95 | 60 | 95 | 60 | 100 | 60 | 100 | 60 |
| C53 | ALQYQVGLTFSPFEKVELSA | 100 | 100 | 100 | 100 | 100 | 100 | 100 | 100 | 100 | 100 | 100 | 100 | 100 | 100 | 100 | 100 | 100 | 100 |
| C54 (Nichols/MexicoA/  UW249) | SPFEKVELSAQWEQGVLADA | 100 | 100 | 100 | 100 | 100 | 100 | 100 | 100 | 90 | 100 | 90 | 100 | 100 | 100 | 90 | 90 | 90 | 90 |
| C54 (Sea81-4/Bal3/  SamoaD/IraqB) | SPFEKVELSAQWEQGVLSDV | 90 | 90 | 90 | 90 | 90 | 90 | 90 | 90 | 100 | 90 | 100 | 90 | 90 | 90 | 100 | 100 | 100 | 100 |
| C55 (Nichols) | QWEQGVLADAPYMGIAESIW | 100 | 100 | 100 | 100 | 100 | 100 | 85 | 100 | 90 | 100 | 90 | 100 | 85 | 100 | 80 | 90 | 90 | 90 |
| C55 (MexicoA/UW249) | QWEQGVLADAPYMGITQSIG | 90 | 90 | 90 | 90 | 90 | 90 | 100 | 85 | 80 | 85 | 80 | 85 | 100 | 85 | 90 | 90 | 80 | 90 |
|  |  | **Percentage Identity with Peptide by Strain and Allele** | | | | | | | | | | | | | | | | | |
|  |  | **Nichols** | | **Chicago** | | **Bal73-1** | | **MexicoA** | | **Sea81-4** | | **Bal3** | | **UW249** | | **SamoaD** | | **IraqB** | |
| **Name** | **Peptide Sequence** | **Tpr**  **C** | **Tpr**  **D** | **Tpr**  **C** | **Tpr**  **D** | **Tpr**  **C** | **Tpr**  **D** | **Tpr**  **C** | **Tpr**  **D** | **Tpr**  **C** | **Tpr**  **D** | **Tpr**  **C** | **Tpr**  **D** | **Tpr**  **C** | **Tpr**  **D** | **Tpr**  **C** | **Tpr**  **D** | **Tpr**  **C** | **Tpr**  **D** |
| C55 (Sea81-4/Bal3/  IraqB) | QWEQGVLSDVPYMGIAESIW | 85 | 85 | 85 | 85 | 85 | 85 | 75 | 85 | 100 | 90 | 100 | 90 | 75 | 90 | 85 | 100 | 100 | 100 |
| C55 (SamoaD) | QWEQGVLSDVPYMGITQSIW | 80 | 80 | 80 | 80 | 80 | 80 | 85 | 90 | 85 | 75 | 85 | 75 | 85 | 80 | 100 | 90 | 90 | 90 |
| C56 (Nichols/Sea81-4/  Bal3/IraqB) | PYMGIAESIWSERHFGTLVCGMKVTW | 100 | 100 | 100 | 100 | 100 | 100 | 85 | 100 | 100 | 100 | 100 | 100 | 85 | 100 | 88 | 100 | 100 | 100 |
| C56 (MexicoA/UW249) | PYMGITQSIGSDRHFGTLVCGMKVTW | 85 | 85 | 85 | 85 | 85 | 85 | 100 | 85 | 85 | 85 | 85 | 85 | 100 | 85 | 92 | 85 | 85 | 85 |
| C56 (SamoaD) | PYMGITQSIWSERHFGTFVCGMKVTW | 88 | 88 | 88 | 88 | 88 | 88 | 88 | 88 | 88 | 88 | 88 | 88 | 88 | 88 | 100 | 88 | 88 | 88 |
| D26 | ALDAGNQHQSNAQFYARMAP | 60 | 60 | 60 | 60 | 60 | 60 | 60 | 100 | 60 | 100 | 60 | 100 | 60 | 100 | 50 | 50 | 50 | 50 |
| D26 (IraqB) | ALDAGNQHQSDTKFYFRMAP | 50 | 50 | 50 | 50 | 50 | 50 | 50 | 50 | 50 | 50 | 50 | 50 | 50 | 50 | 50 | 100 | 50 | 100 |
| D27 | QFYARMAPSQRVHEVITSLG | 10 | 10 | 10 | 10 | 10 | 10 | 10 | 100 | 10 | 100 | 10 | 100 | 10 | 100 | 10 | 85 | 10 | 85 |
| D27 (IraqB) | KFYFRMAPSQRVHEVITSLG | 10 | 10 | 10 | 10 | 10 | 10 | 10 | 85 | 10 | 85 | 10 | 85 | 10 | 85 | 10 | 95 | 10 | 100 |
| D28 | RVHEVITSLGDTLLTSPQQD | 15 | 15 | 15 | 15 | 15 | 15 | 15 | 100 | 15 | 100 | 15 | 100 | 15 | 100 | 15 | 95 | 15 | 95 |
| D29 | DTLLTSPQQDVVSFFVQELS | 10 | 10 | 10 | 10 | 10 | 10 | 10 | 100 | 10 | 100 | 10 | 100 | 10 | 100 | 10 | 100 | 10 | 100 |
| D30 | VVSFFVQELSKGSLLEKAGL | 25 | 25 | 25 | 25 | 25 | 25 | 25 | 100 | 25 | 100 | 25 | 100 | 25 | 100 | 25 | 100 | 25 | 100 |
| D31 | KGSLLEKAGLVTLLAQRTIV | 35 | 35 | 35 | 35 | 35 | 35 | 35 | 100 | 35 | 100 | 35 | 100 | 35 | 100 | 35 | 100 | 35 | 100 |
| D32 | VTLLAQRTIVGLASSGGYLR | 20 | 20 | 20 | 20 | 20 | 20 | 20 | 100 | 20 | 100 | 20 | 100 | 20 | 100 | 20 | 100 | 20 | 100 |
| D33 | GLASSGGYLRHLNGKGLEIN | 10 | 10 | 10 | 10 | 10 | 10 | 10 | 100 | 10 | 100 | 10 | 100 | 10 | 100 | 10 | 100 | 10 | 100 |
| D34 | HLNGKGLEINMRLIEQQKNP | 5 | 5 | 5 | 5 | 5 | 5 | 5 | 100 | 5 | 100 | 5 | 100 | 5 | 100 | 5 | 100 | 5 | 100 |
| D35 | MRLIEQQKNPDARMRTALFI | 20 | 20 | 20 | 20 | 20 | 20 | 20 | 100 | 20 | 100 | 20 | 100 | 20 | 100 | 20 | 100 | 20 | 100 |
| D36 | DARMRTALFISWLQFTYTKT | 25 | 25 | 25 | 25 | 25 | 25 | 25 | 100 | 25 | 100 | 25 | 100 | 25 | 100 | 25 | 100 | 25 | 100 |
| D37 | ALFISSWLQFTYTKTLNIDAL | 10 | 10 | 10 | 10 | 10 | 10 | 10 | 100 | 10 | 100 | 10 | 100 | 10 | 100 | 10 | 100 | 10 | 100 |
| D38 | YTKTLNIDALLRMQWRWLSS | 75 | 75 | 75 | 75 | 75 | 75 | 75 | 80 | 75 | 80 | 75 | 80 | 75 | 80 | 80 | 80 | 75 | 75 |
| D39 | LRMQWRWLSSGIYFATAGTN | 95 | 95 | 95 | 95 | 95 | 95 | 95 | 100 | 95 | 100 | 95 | 100 | 95 | 100 | 95 | 95 | 95 | 95 |
| D40 | GIYFATAGTNIFGERVFFKN | 80 | 80 | 80 | 80 | 80 | 80 | 80 | 100 | 80 | 100 | 80 | 100 | 80 | 100 | 80 | 100 | 80 | 100 |
| D41 | IFGERVFFKNQADHFDFAGF | 65 | 65 | 65 | 65 | 65 | 65 | 65 | 100 | 65 | 100 | 65 | 100 | 65 | 100 | 65 | 100 | 65 | 100 |
| D42 | QADHFDFAGFLKLETKSGDP | 80 | 80 | 80 | 80 | 80 | 80 | 80 | 100 | 80 | 100 | 80 | 100 | 80 | 100 | 80 | 100 | 80 | 100 |
| D45 | GVEARVYIPLTYIFYINNGG | 90 | 90 | 90 | 90 | 90 | 90 | 90 | 100 | 90 | 100 | 90 | 100 | 90 | 100 | 90 | 100 | 90 | 100 |
| D46 | TYIFYINNGGAQYKGSNSDG | 45 | 45 | 45 | 45 | 45 | 45 | 45 | 100 | 45 | 100 | 45 | 100 | 45 | 100 | 45 | 100 | 45 | 100 |
| D47 | AQYKGSNSDGVINTPILSKA | 50 | 50 | 50 | 50 | 50 | 50 | 50 | 100 | 50 | 100 | 50 | 100 | 50 | 100 | 50 | 100 | 50 | 100 |
| D48 | VINTPILSKAWCSYRIPLGS | 90 | 90 | 90 | 90 | 90 | 90 | 90 | 100 | 90 | 100 | 90 | 100 | 90 | 100 | 90 | 100 | 90 | 100 |
| D50 | HAWLAPHTSVLWATNRFNHN | 80 | 80 | 80 | 80 | 80 | 80 | 80 | 100 | 80 | 100 | 80 | 100 | 80 | 100 | 80 | 100 | 80 | 100 |
| D51 | LWATNRFNHNQSGDALLREH | 45 | 45 | 45 | 45 | 45 | 45 | 45 | 100 | 45 | 100 | 45 | 100 | 45 | 100 | 40 | 100 | 40 | 100 |
| D52 | QSGDALLREHALQYQVGLTF | 65 | 65 | 65 | 65 | 65 | 65 | 65 | 100 | 65 | 100 | 65 | 100 | 65 | 100 | 60 | 100 | 60 | 100 |

*In Figure 2-5, When necessary, strain names are abbreviated as follows: N: Nichols; M: MexicoA; Sea: Sea81-4; B: Bal3; U: UW249; S: SamoaD; I: IraqB.

“C” indicates peptides mapping to conserved portions of the TprC/D/D_2_ proteins. “D” peptides map to TprD_2_ central region, which greatly differ from TprC/D variants. Peptides are numbered sequentially (e.g. C1 shares the last 10 aa with C2, C2 shares the last 10 aa with C3 and so forth). If multiple peptides share the same name (e.g. C47) the strain name (Table 1) or abbreviation (Figures) follows the peptide number.
