# Supplementary material for: B-Cell Epitope Mapping of TprC and TprD Variants of Treponema pallidum Subspecies Informs Vaccine Development for Human Treponematoses
Source: Front Immunol. 2022 Mar 29;13:862491. doi: 10.3389/fimmu.2022.862491 (PMC9001972; doi:10.3389/fimmu.2022.862491)
Supplement: Supplementary file 3 [file Table_3.docx]

| **Table S7.** Sequence of reactive peptides identified by infected-rabbit sera | | | | | |  |  |
| --- | --- | --- | --- | --- | --- | --- | --- |
| Sequences of reactive peptides based on Fig.2A (subspecies *pallidum*) | | | | | | | |
| **Peptide or peptide range** | **Experimentally determined Epitope-containing sequence** | **Location per AlphaFold** | | **B-cell epitope predicted by** | | | |
|  |  |  |  | IEDB | | BCpreds | BepiPred2.0 |
| C1-C3 | GVLTPQVSGTAQLQWGIAFQ  KNPRTGPGKHTHGFRTTNSL | Scaffold (C1) and ExL1 (C2-3) | | X | | X | X |
| C6 | THTRRGEARSGVWAQLQLKD | Scaffold | | X | | X | X |
| C13-C14 | KPFVTRAYSEKDTRYAPGFSGSGAKLGYQA | ExL3 | | X | | X |  |
| C18 | GAWDSTDTTHSKYGFGADAT | ExL4 | | X | | X | X |
| C20 | LSYGVDRQRLLTLELAGNAT | Scaffold | | X | |  |  |
| C25-C29 | EPGAGFRFSFALDAGNQHQS  NAHAQTQERAILKAREVFRR  VEGKLVQNLPNIMMPPGITE | Scaffold (C25) and ExL6 (C26-C29) | | X | | X | X |
| C39 | LRMQWKWLSSGIYFATAGTN | Scaffold | |  | | None |  |
| C43 | LKLETKSGDPYTHLLTGLNA | Scaffold | | X | | X | X |
| N–C46-47 | TYIRYRNNGGYELNGAVPPGTINMPILGKA | ExL8 | | X | |  |  |
| C50-C51 | HAWLAPHTSVLGTTNRFNIINAAGNLLNER | ExL9 | | X | | X | X |
| C53-C55 | ALQYQVGLTFSPFEKVELSA  QWEQGVL**S/A**D**V/A**PYMGIAESIW (N) or  QWEQGVLSDVPYMGIAESIW (Sea81-4/Bal3)* | Scaffold (C53) and ExL10 (C54-C55) | | X | | X | X |
| Sequences of reactive peptides based on Fig.2B (subspecies *pertenue* and *endemicum*) | | | | | | | |
| C1 | GVLTPQVSGTAQLQWGIAFQ | Scaffold | |  | | None |  |
| C6 | THTRRGEARSGVWAQLQLKD | Scaffold | |  | | X | X |
| C13-C14 | KPFVTRAYSEKDTRYAPGFSGSGAKLGYQA | ExL3 | | X | | X | X |
| C18 | GAWDSTDTTHSKYGFGADAT | ExL4 | | X | | X |  |
| C20 | LSYGVDRQRLLTLELAGNAT | Scaffold | | X | |  |  |
| S – C22 | LEQHYRKGTEDSTNENKTAL | ExL5 | | X | | X |  |
| C25 | EPGAGFRFSFALDAGNQHQS | Scaffold | |  | | X |  |
| C33 | SAIQTVLAAGALAALVSQLV | ExL6 | | X | |  |  |
| C36 | FRSSDPRVVTAKLLAFLERA | ExL6 | |  | | None |  |
| C43 | LKLETKSGDPYTHLLTGLNA | Scaffold | | X | | X |  |
| S – C46 | TYVFYKNNGGYPLNGVVPSG | ExL8 | | X | | X | X |
| I – C46 | TYVFYKNNGGYELNGVVPPG | ExL8 | | X | | X | X |
| S – C47 | YPLNGVVPSGTINMPILGKA | ExL8 | | X | | X | X |
| I – C47 | YELNGVVPPGIINMPILGKA | ExL8 | | X | | X | X |
| C51 | LGTTNRFNIINAAGNLLNER | ExL9 | | X | | X | X |
| C53 | ALQYQVGLTFSPFEKVELSA | Scaffold | |  | | None |  |
| S – C54 | SPFEKVELSAQWEQGVLSDV | ExL10 | | X | | X | X |
| I – C55 | QWEQGVLSDVPYMGIAESIW | ExL10 | | X | | X | X |
| Sequences of reactive peptides based on Fig.2C | | | | | | | |
| D34-D36 | HLNGKGLEINMRLIEQQKNP  DARMRTALFISWLQFTYTKT | | ExL6 | | X | X | X |
| I – C39 | LRMQWKWLSSGIYFATAGTN | | Scaffold | |  | None |  |
| C43 | LKLETKSGDPYTHLLTGLNA | | Scaffold | | X | X | X |
| D46-D47 | TYIFYINNGGAQYKGSNSDGVINTPILSKA | | ExL8 | | X | X | X |
| D49 | WCSYRIPLGSHAWLAPHTSV | | Scaffold | |  | None |  |
| D51 | LWATNRFNHNQSGDALLREH | | ExL9 | | X | X | X |

Lightly shaded peptides are in the NH_2_-terminal portion of the protein.

*S and V in the Sea 81-4/Bal3 strain, A and A in Nichols
