# Supplementary material for: B-Cell Epitope Mapping of TprC and TprD Variants of Treponema pallidum Subspecies Informs Vaccine Development for Human Treponematoses
Source: Front Immunol. 2022 Mar 29;13:862491. doi: 10.3389/fimmu.2022.862491 (PMC9001972; doi:10.3389/fimmu.2022.862491)
Supplement: Supplementary file 4 [file Table_4.docx]

| **Table S8.** Sequences of reactive peptides following rabbit immunization | | |
| --- | --- | --- |
| Sequences of reactive peptides based on results shown in Fig.4A.  Immunization with TprC full-length Nichols variant | | |
| **Peptide or peptide range** | **Experimentally determined Epitope-containing sequences** | **Location per AlphaFold** |
| C1-C3 | GVLTPQVSGTAQLQWGIAFQ  KNPRTGPGKHTHGFRTTNSL | Scaffold (C1) and ExL1 (C2-C3) |
| C5-C6 | TISLPLVSKHTHTRRGEARS THTRRGEARSGVWAQLQLKD | Scaffold |
| **C9^1^** | **STALSFTKPTASFQATLHCY** | ExL2/Scaffold |
|  |  |  |
| C16-18 | HNVGNSGVDVDIGFLSFLSN  GAWDSTDTTHSKYGFGADAT | Scaffold (c16-17)/ExL4 (C17-18)/Scaffold (C18) |
| C28 | ILKAREVFRRVEGKLVQNLP | ExL6 |
| C32 | AALIAEGTLGSAIQTVLAAG | ExL6 |
| C35 | PNIEQGVRDVFRSSDPRVVT | ExL6 |
| N/Sea/U - C47 | YELNGAVPPGTINMPILGKA | ExL8 |
| C53 | ALQYQVGLTFSPFEKVELSA | Scaffold |
| C55 | QWEQGVLSDVPYMGIAESIW | ExL10 |
| Sequences of reactive peptides based on results shown in Fig.4B.  Immunization with TprC full-length Bal3 variant | | |
| C1-C3 | GVLTPQVSGTAQLQWGIAFQ  KNPRTGPGKHTHGFRTTNSL | Scaffold (C1) and ExL1 (C2-C3) |
| C6-C7 | THTRRGEARSGVWAQLQLKDLAVELASSKS | Scaffold (C6) and ExL2 (C7) |
| C13-C14 | KPFVTRAYSEKDTRYAPGFSGSGAKLGYQA | ExL3 |
| **C16^1^**-C18 | **HNVGNSGVDVDIGFLSFLSN** GAWDSTDTTHSKYGFGADAT | Scaffold (C16) and ExL4 (C17-C18) |
| C20 | LSYGVDRQRLLTLELAGNAT | Scaffold |
| C43 | LKLETKSGDPYTHLLTGLNA | Scaffold |
| C47 | YELNRVVPSGIINMPILGKA | ExL8 |
| C49 | WCSYRIPLGSHAWLAPHTSV | Scaffold |
| C51 | LGTTNRFNIINAAGNLLNER | ExL9 |
| Sequences of reactive peptides based on results shown in Fig.4C.  Immunization with TprC full-length MexicoA variant | | |
| C1-C3 | GVLTPQVSGTAQLQWGIAFQ  KNPRTGPGKHTHGFRTTNSL | Scaffold (C1) and ExL1 (C2-C3) |
| C5-C6 | TISLPLVSKHTHTRRGEARS THTRRGEARSGVWAQLQLKD | Scaffold |
| **C9^1^** | **STALSFTKPTASFQATLHCY** | Scaffold/ExL2 |
| C28 | ILKAREVFRRVEGKLVQNLP | ExL6 |

**^1^** C9/C16 peptides were not predicted to harbor B-cell

epitopes by IEDB, BCpreds and bepiPred2.0
