# Supplementary material for: B-Cell Epitope Mapping of TprC and TprD Variants of Treponema pallidum Subspecies Informs Vaccine Development for Human Treponematoses
Source: Front Immunol. 2022 Mar 29;13:862491. doi: 10.3389/fimmu.2022.862491 (PMC9001972; doi:10.3389/fimmu.2022.862491)
Supplement: Supplementary file 5 [file Table_5.docx]

**Table S9. Treponemal strains used in this study**

| **Species,**  **subspecies** | **Strain name** | **Source** | **Location** | **Year of isolation** |
| --- | --- | --- | --- | --- |
| *Tp. pallidum* | Nichols^a^ | Cerebrospinal fluid | Washington DC | 1912 |
|  | Sea81-4^b^ | Primary chancre | Seattle | 1980 |
|  | Bal3^c^ | Blood, congenital | Baltimore | Unknown |
|  | MexicoA^c^ | Primary chancre | Mexico | 1953 |
|  | Street14^d^ | Skin | Atlanta | 1977 |
|  | Bal73-1^c^ | Aqueous humor, congenital | Baltimore | 1973 |
|  | UW249C^e^ | Cerebrospinal fluid | Seattle | 2004 |
| *Tp. endemicum* | IraqB^c^ | Oral mucous patches | Iraq | 1951 |
| *Tp. pertenue* | SamoaD^c^ | Skin lesion | Western Samoa | 1953 |

^a^ Originally provided by James N. Miller, University of California, Los Angeles, CA.

^b^ Strain isolated in Seattle by Sheila A. Lukehart, University of Washington, Seattle, WA.

^c^ Strains provided by Paul Hardy and Ellen Nell, Johns Hopkins University, Baltimore, MD.

^d^ Provided by Sandra A. Larsen, Center for Disease Control and Prevention, Atlanta, GA.

^e^ Provided by Christina Marra, University of Washington, Seattle, WA.
