# Supplementary material for: B-Cell Epitope Mapping of TprC and TprD Variants of Treponema pallidum Subspecies Informs Vaccine Development for Human Treponematoses
Source: Front Immunol. 2022 Mar 29;13:862491. doi: 10.3389/fimmu.2022.862491 (PMC9001972; doi:10.3389/fimmu.2022.862491)
Supplement: Supplementary file 6 [file Table_6.docx]

**Table S2. TprC – IEDB B-cell epitope prediction results**

| **No.** | **Start** | **End** | **Peptide** | **Peptide(s)** | **Location** | **Length** |
| --- | --- | --- | --- | --- | --- | --- |
| 1 | 41 | 59 | FQKNPRTGPGKHTHGFRTT | C2-C3 | ExL1 | 19 |
| 2 | 71 | 82 | KHTHTRRGEARS | C5-C6 | Scaffolding | 12 |
| 3 | 95 | 113 | VELASSKSSTALSFTKPTA | C7-C9 | ExL2 | 19 |
| 4 | 132 | 180 | PSCVVNFAQLWKPFVTRAYSEKDTRYAPGFSGSGAKLGYQAHNVGNSGV | C11-C16 | ExL3 | 49 |
| 5 | 192 | 208 | NGAWDSTDTTHSKYGFG | C17-C18 | ExL4 | 17 |
| 6 | 215 | 220 | YGVDRQ | C19-C20 | Scaffolding | 6 |
| 7 | 233 | 251 | LDQNYVKGTEDSKNENKTA | C21-C23 | ExL5 | 19 |
| 8 | 277 | 295 | GNQHQSNAHAQTQERAILK | C26-C27 | ExL6 | 19 |
| 9 | 309 | 321 | QNLPNIMMPPGIT | C29 | ExL6 | 13 |
| 10 | 343 | 352 | SAIQTVLAAG | C32-C33 | ExL6 | 10 |
| 11 | 359 | 377 | SQLVPNIEQGVRDVFRSSD | C34-C36 | ExL6 | 19 |
| 12 | 393 | 396 | PMNA | C37-C38 | ExL6 | 4 |
| 13 | 421 | 436 | TNIFGKRVFATTRAHY | C40 | ExL7 | 16 |
| 14 | 448 | 452 | KSGDP | C42-C43 | Scaffolding | 5 |
| 15 | 476 | 493 | FYRNNGGYELNRVVPSGI | C45-C47 | ExL8 | 18 |
| 16 | 527 | 539 | NRFNIINAAGNLL | C50-C51 | ExL9 | 13 |
| 17 | 564 | 585 | WEQGVLSDVPYMGIAESIWSER | C54-C56 | ExL10 | 22 |

**Table S3. TprD_2_ – IEDB B-cell epitope prediction results**

| **No.** | **Start** | **End** | **Peptide** | **Peptide(s)** | **Location** | **Length** |
| --- | --- | --- | --- | --- | --- | --- |
| 1 | 41 | 59 | FQKNPRTGPGKHTHGFRTT | C2-C3 | ExL1 | 19 |
| 2 | 71 | 82 | KHTHTRRGEARS | C5-C6 | Scaffolding | 12 |
| 3 | 95 | 112 | VELASSKSSTALSFTKPT | C7-C9 | ExL2 | 18 |
| 4 | 132 | 180 | PSCVVNFAQLWKPFVTRAYSEKDTRYAPGFSGSGAKLGYQAHNVGNSGV | C11-C16 | ExL3 | 49 |
| 5 | 192 | 208 | NGAWDSTDTTHSKYGFG | C17-C18 | ExL4 | 17 |
| 6 | 215 | 220 | YGVDRQ | C19-C20 | Scaffolding | 6 |
| 7 | 233 | 255 | LDQNYVKGTEDSKNENKTALLWG | C21-C23 | ExL5 | 23 |
| 8 | 277 | 297 | GNQHQSNAQFYARMAPSQRVH | D26-D27 | ExL6 | 21 |
| 9 | 308 | 316 | LTSPQQDVV | D29 | ExL6 | 9 |
| 10 | 320 | 332 | VQELSKGSLLEKA | D30 | ExL6 | 13 |
| 11 | 339 | 362 | AQRTIVGLASSGGYLRHLNGKGLE | D32-D34 | ExL6 | 24 |
| 12 | 366 | 376 | RLIEQQKNPDA | D34-D35 | ExL6 | 11 |
| 15 | 419 | 434 | TNIFGERVFFKNQADH | D40 | ExL7 | 16 |
| 16 | 446 | 450 | KSGDP | C42-C43 | Scaffolding | 5 |
| 17 | 475 | 490 | YINNGGAQYKGSNSDG | D45-D46 | ExL8 | 16 |
| 18 | 525 | 537 | NRFNHNQSGDALL | C50-C51 | ExL9 | 13 |
| 19 | 562 | 570 | WEQGVLADA | C54-C55 | ExL10 | 9 |
| 20 | 578 | 583 | SIWSER | C56 | ExL10 | 6 |

**Table S4. TprC – FBCPred B-cell epitope prediction results**

| **Position** | **Epitope** | **Peptide(s)** | **Location** | **Score** |
| --- | --- | --- | --- | --- |
| 42 | QKNPRTGPGKHTHG | C2-C3 | ExL1 | 0.999 |
| 70 | SKHTHTRRGEARSG | C5-C6 | Scaffolding | 0.895 |
| 105 | ALSFTKPTASFQAT | C8-C9 | ExL2 | 0.962 |
| 130 | TSPSCVVNFAQLWK | C11-C12 | ExL3 | 0.985 |
| 156 | RYAPGFSGSGAKLG | C14-C15 | ExL3 | 0.998 |
| 171 | QAHNVGNSGVDVDI | C15-C16 | Scaffolding | 0.992 |
| 191 | SNGAWDSTDTTHSK | C17-C18 | ExL4 | 0.995 |
| 241 | TEDSKNENKTALLW | C22-C23 | ExL5 | 0.923 |
| 259 | RLTLEPGAGFRFSF | C24-C25 | Scaffolding | 1 |
| 308 | VQNLPNIMMPPGIT | C29 | ExL6 | 0.809 |
| 362 | VPNIEQGVRDVFRS | C34-C35 | ExL6 | 0.935 |
| 441 | GFLKLETKSGDPYT | C42-C43 | Scaffolding | 0.997 |
| 464 | VEARVYIPLTYVFY | C45 | ExL8 | 0.825 |
| 524 | GTTNRFNIINAAGN | C51-C52 | ExL9 | 0.996 |
| 561 | SAQWEQGVLSDVPY | C54-C55 | ExL10 | 0.919 |

**Table S5. TprD_2_ – FBCPred B-cell epitope prediction results**

| **Position** | **Epitope** | **Peptide(s)** | **Location** | **Score** |
| --- | --- | --- | --- | --- |
| 42 | QKNPRTGPGKHTHG | C2-C3 | ExL1 | 0.999 |
| 70 | SKHTHTRRGEARSG | C5-C6 | Scaffolding | 0.895 |
| 105 | ALSFTKPTASFQAT | C8-C9 | ExL2 | 0.962 |
| 130 | TSPSCVVNFAQLWK | C11-C12 | ExL3 | 0.985 |
| 156 | RYAPGFSGSGAKLG | C14-C15 | ExL3 | 0.998 |
| 171 | QAHNVGNSGVDVDI | C15-C16 | Scaffolding | 0.992 |
| 191 | SNGAWDSTDTTHSK | C17-C18 | ExL4 | 0.995 |
| 241 | TEDSKNENKTALLW | C22-C23 | ExL5 | 0.923 |
| 259 | RLTLEPGAGFRFSF | C24-C25 | Scaffolding | 1 |
| 308 | LTSPQQDVVSFFVQ | D29 | ExL6 | 0.8 |
| 365 | MRLIEQQKNPDARM | D34-D35 | ExL6 | 0.829 |
| 439 | GFLKLETKSGDPYT | D42 | Scaffolding | 0.997 |
| 462 | VEARVYIPLTYIFY | D45-D46 | ExL8 | 0.975 |
| 483 | YKGSNSDGVINTPI | D47 | ExL8 | 0.996 |
| 523 | ATNRFNHNQSGDAL | D51-D52 | ExL9 | 0.956 |
| 558 | LSAQWEQGVLADAP | C54-C55 | ExL10 | 0.895 |

**Fig. S1. TprC and TprD_2_ BepiPred B-cell epitope prediction results**

**(A)**

#
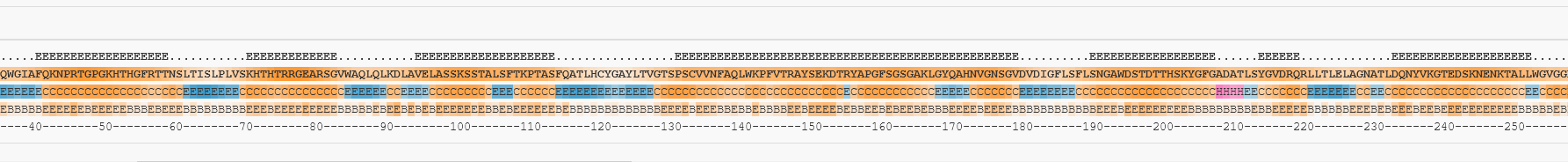


#
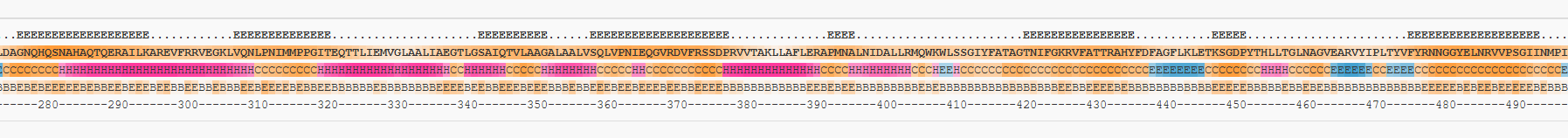


#
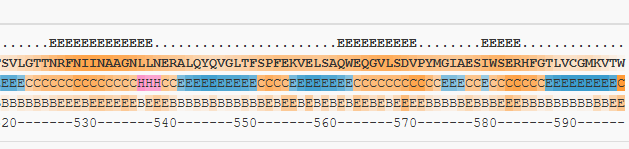


**(B)**


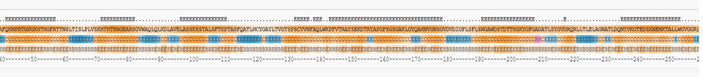


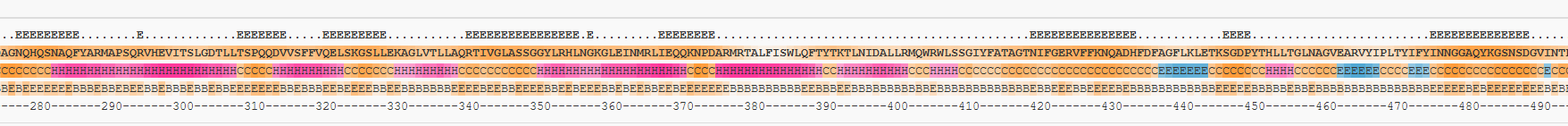


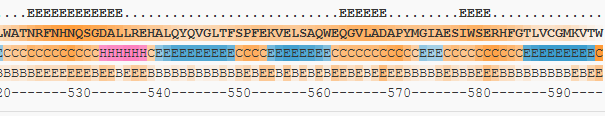


**Fig.S1 Legend**

The output format shows the BepiPred-2.0 predictions and epitope classification for TprC (**A**) and TprD_2_ (**B**). The BepiPred-2.0 predictions are used to set the background color of the protein sequences. All predictions greater than a user-defined threshold (by default 0.5) are marked as ‘E’ in the ‘Epitopes’ line above the protein sequence itself.

**Fig.S3. pLDDT analysis for all TprC (A) and TprD_2_ (B) models generated by AlphFold2**


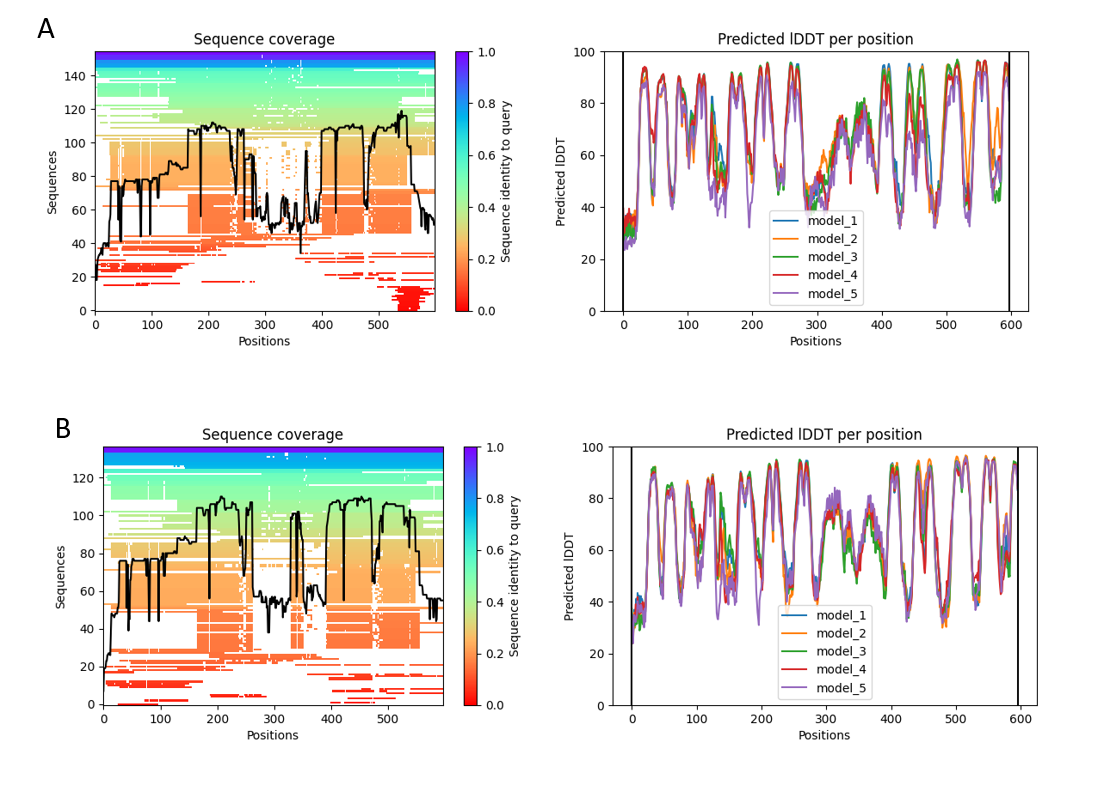


**Fig.S3 Legend**

pLDDT analysis for all TprC (**A**) and TprD_2_ (**B**) models generated by AlphFold2.
